# Synchronous Bilateral Multifocal Warthin's Tumor Mimicking Primary Malignancy on ^18^ F-FDG Whole Body PET/CT in a Case of Metastatic Cervical Lymph Nodes from Unknown Primary Malignancy

**DOI:** 10.1055/s-0042-1750401

**Published:** 2022-09-09

**Authors:** Shyma Basheer, Alamelu Satish, Archana George Vallonthaiel, Manjit Sarma

**Affiliations:** 1 Department of Nuclear Medicine & Molecular Imaging, Amrita Institute of Medical Sciences, Kochi, Kerala, India

**Keywords:** Warthin's tumor, metastatic cervical lymph node with unknown origin, ^18^ F-FDG, PET/CT

## Abstract

Warthin's tumor is a benign and frequently encountered salivary gland neoplasm. Bilaterality and multifocality are rare in Warthin's tumor. Synchronous cervical lymph nodal metastasis with unknown primary in a case of Warthin's tumor can raise a suspicion of primary malignancy of the parotid gland. We present a case of bilateral multifocal Warthin's tumor with synchronous squamous cell carcinoma metastasis to the cervical lymph node. ^18^ F-fluorodeoxyglucose whole body positron emission tomography/computed tomography imaging showed hypermetabolic bilateral multifocal parotid lesions and metastatic cervical lymph node with unknown primary malignancy.

## Key Messages

- Warthin's tumor shows intense FDG uptake on ^18^ F-FDG whole body PET/CT and can be multifocal and bilateral.
- Coexistent cervical lymph nodal metastasis should be investigated thoroughly for aerodigestive malignancies and can also present as malignancy of unknown origin.

## Introduction

Warthin's tumor is the second most common parotid gland tumor after pleomorphic adenoma. Although unifocal Warthin's tumor is common, multifocality and bilaterality are rare. Squamous cell carcinoma cervical lymph nodal metastasis in a patient with Warthin's tumor can raise a suspicion for primary malignancy in the parotid gland. This is a unique case report of metastatic cervical lymph node and ^18^ F-fluorodeoxyglucose ( ^18^ F-FDG) whole body positron emission tomography/computed tomography (PET/CT) showed metabolically active bilateral parotid gland lesions and a right cervical lymph node with no possible primary site of malignancy.

## Case History

**Fig. 1:**
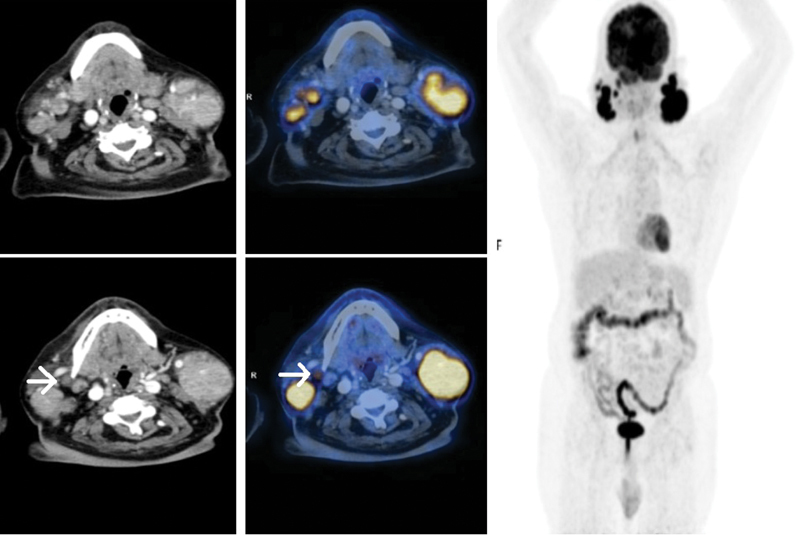
Axial section on computed tomography (CT), positron emission tomography (PET), and fused PET-CT showing intense fluorodeoxyglucose (FDG) uptake in the parotid region corresponding to multiple enhancing nodules in both lobes of parotid gland on axial CT ( *above* ) and FDG avid right level II lymph node ( *below* ). ^18^ F-FDG PET maximal intensity projection image shows multiple foci of increased abnormal uptake of FDG seen bilaterally in the neck. Minimal tracer uptake in the right lobe of thyroid gland and diffuse uptake in stomach walls are also seen, with no corresponding lesions on CT images. Physiological tracer uptake is seen in the brain, kidneys, and urinary bladder.

**Fig. 2:**
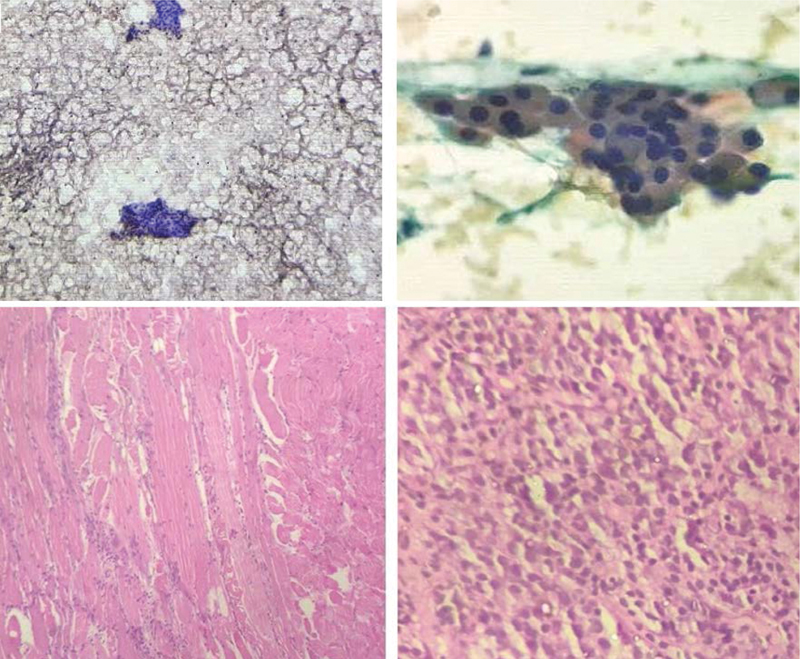
Micrograph of cytology from bilateral parotid swellings showing monolayer cells of oncocytic epithelium with round nucleus and inconspicuous nucleoli with background of lymphocytes and proteinaceous material which is suggestive of Warthin's tumor ( *above and below left* ). Specimen from right cervical lymph node ( *above right* ) shows atypical squamous cells with orangeophilic cytoplasm round to angular hyperchromatic nucleus suggestive of squamous cell carcinoma ( *below right* ).

A 70-year-old male, a chronic smoker, presented with complaints of swelling of the cheek for the last 6 years. Ultrasound showed hyperechoic well-defined lesions with similar echotexture and vascularity seen in both lobes of parotid gland and enlarged lymph nodes in bilateral level II nodal stations. Ultrasound-guided fine-needle aspiration (FNA) from bilateral parotid gland was suggestive of Warthin's tumor. However, right cervical lymph node showed well-differentiated squamous cell carcinoma. Indirect laryngoscopy and upper gastrointestinal endoscopy were normal. ^18^ F-FDG whole body PET/CT imaging ( Fig. 1 ) showed metabolically active multiple metabolically active lesions in the bilateral parotid gland and metabolically active bilateral level II lymph nodes. No other possible site of primary malignancy was seen. Minimal tracer uptake was seen in the right lobe of thyroid gland and diffuse uptake in stomach walls, with no corresponding lesions on CT images. No suspicious nodules or significant mediastinal lymph nodes were seen in lung parenchyma on inspiratory lung CT images. Patient subsequently underwent repeat biopsy from the right parotid lesion having the maximum standardized uptake value (14.6). The repeat biopsy( Fig. 2 ) was also suggestive of Warthin's tumor. The patient subsequently underwent radiotherapy for cervical lymph nodal metastasis.

## Discussion

Warthin's tumor is benign neoplasm of the major salivary glands. It is the second most common parotid gland tumor after pleomorphic adenoma accounting for approximately 15% of all parotid epithelial tumors. 1 Unilateral metabolically active lesion of parotid gland on PET/CT imaging can occur from a large spectrum of benign or malignant, such as adenoma, Warthin's tumor, oncocytic neoplasm, lymphoma, and primary or secondary malignancy of the gland. Bilateral multifocal Warthin's tumor is less common. 2

Metastatic cervical lymph node with unknown primary malignancy is a metastatic disease in the lymph nodes of the neck without any evidence of a primary tumor after appropriate investigation. In patient initially presenting with cervical lymph nodal metastasis, 2 to 10% patients account for the unknown primary malignancy after a routine clinical workup. 3

The pathophysiology that allows a primary tumor to remain hidden after the development of metastases is yet not known. Various theories include regression or involution of the primary and development of the malignancy of unknown origin in stem cells with capacity to differentiate into multiple cell lines to the liver, muscles, skin, or even the cells of the gastrointestinal tract.

Proper recognition of clinical presentation, CT or magnetic resonance imaging and PET/CT, may guide to the diagnosis of primary malignancy. Direct laryngoscopy and careful endoscopy of the nasopharynx and biopsy of the suspicious areas are also warranted. Ultrasound-guided FNA or histologic biopsy from the initial occult site should be obtained for initial tissue diagnosis. Histologic biopsy should be obtained, preferably from the occult primary site. Tonsillectomy and lingual tonsillectomy may also be indicated to identify a possible primary site. Immunohistochemistry is used to define the cell differentiation and identify the primary tumor in approximately 25 to 30% cases. Squamous cell carcinoma is the most common cytopathological finding in metastatic cervical lymph nodes. Salivary gland cancers, well-differentiated thyroid malignancies, and nonsquamous malignancies originating from skin are the other common histologies in the neck. Metastasis to cervical lymph nodes is developed more often from the upper aerodigestive tract primary malignancies. 3 4

As Warthin's tumor is more commonly seen in smokers, it may be mistaken for malignancy when associated aerodigestive tract cancer is present. The clinicians should be aware of the possibility of Warthin's tumor masquerading as malignancy in these patients. Further evaluation by FNA, ultrasound-guided FNA, or technetium-99 salivary scintigraphy can confirm the diagnosis and, in such cases, surgery can be avoided for the benign lesion. 5

The studies on FDG PET/CT in salivary gland tumors are limited. Although Warthin's tumor is of benign nature, it has been shown to concentrate FDG with uptake similar to the malignant salivary gland tumors, thereby reducing the sensitivity of PET to discriminate benign and malignant tumors. 6 This case after PET/CT imaging, a repeat FNA from the most metabolically active lesion in the parotid gland, was suggestive of Warthin's tumor.

Squamous cell carcinoma occurring de novo from the parotid gland is a rare cancer comprising of less than 1% of all salivary gland neoplasms. 7 But in this case of cervical nodal biopsy showing squamous cell carcinoma, hypermetabolic activity in Warthins's tumor with no lesions elsewhere can be mistaken for primary malignancy and should be excluded.

In conclusion, the coexistence of bilateral multifocal Warthin's tumor with cervical lymph nodal metastasis can misguide the clinician to suspect primary malignancy from the salivary gland. In a smoker, where incidence of Warthin's tumor and aerodigestive malignancies are more often, the synchronous presentation of bilateral multifocal Warthin's tumor and metastasis of unknown origin can exist together and complete clinical examination and investigations should be performed to identify the primary site of malignancy.

## References

[1] Chulam T C Noronha Francisco A L Goncalves Filho J Pinto Alves C A Kowalski L P Warthin's tumour of the parotid gland: our experience Acta Otorhinolaryngol Ital 2013 33 06 393 397 24376295 PMC3870448

[2] Nguyen V X Nguyen B D Ram P C Bilateral and multifocal Warthin's tumors of parotid glands: PET/CT imaging Clin Nucl Med 2012 37 02 175 177 22228345 10.1097/RLU.0b013e318238f244

[3] Karapolat I Kumanlıoğlu K Impact of FDG-PET/CT for the detection of unknown primary tumours in patients with cervical lymph node metastases Mol Imaging Radionucl Ther 2012 21 02 63 68 23487242 10.4274/Mirt.344 PMC3590973

[4] Qaseem A Usman N Jayaraj J S Janapala R N Kashif T Cancer of unknown primary: a review on clinical guidelines in the development and targeted management of patients with the unknown primary site Cureus 2019 11 09 e5552 10.7759/cureus.5552 31695975 PMC6820325

[5] Rassekh C H Cost J L Hogg J P Hurst M K Marano G D Ducatman B S Positron emission tomography in Warthin's tumor mimicking malignancy impacts the evaluation of head and neck patients Am J Otolaryngol 2015 36 02 259 263 25523505 10.1016/j.amjoto.2014.11.008

[6] Dua S G Purandare N C Shah S Rangarajan V Bilateral synchronous and multifocal Warthin's tumor mimicking metastases from lung cancer: A rare cause of false positive flourodeoxy glucose positron emission tomography/computed tomography Indian J Nucl Med 2012 27 02 139 140 23723595 10.4103/0972-3919.110717 PMC3665148

[7] Akhtar K Ray P S Sherwani R Siddiqui S Primary squamous cell carcinoma of the parotid gland: a rare entity BMJ Case Rep 2013 2013 bcr2013009467 10.1136/bcr-2013-009467 PMC3669887 23682090

